# Thirty years of heart transplantation at the University Medical Centre Utrecht

**DOI:** 10.1007/s12471-017-0969-0

**Published:** 2017-02-28

**Authors:** A. Sammani, A. M. Wind, J. H. Kirkels, C. Klöpping, M. P. Buijsrogge, F. Z. Ramjakhan, F. W. Asselbergs, N. de Jonge

**Affiliations:** 1 Department of Cardiology, UMC Utrecht, Utrecht, The Netherlands; 2 Department of Cardiothoracic Surgery, UMC Utrecht, Utrecht, The Netherlands

**Keywords:** Heart transplantation, HTx, LVAD, Left ventricular assist device, Demography, Utrecht

## Abstract

**Purpose:**

To analyse patient demographics, indications, survival and donor characteristics for heart transplantation (HTx) during the past 30 years at the University Medical Centre Utrecht (UMCU).

**Methods:**

Data have been prospectively collected for all patients who underwent HTx at the UMCU from 1985 until 2015. Patients who were included underwent orthotopic HTx at an age >14 years.

**Results:**

In total, 489 hearts have been transplanted since 1985; 120 patients (25%) had left ventricular assist device (LVAD) implantation prior to HTx. A shift from ischaemic heart disease to dilated cardiomyopathy has been seen as the leading indication for HTx since the year 2000. Median age at HTx was 49 years (range 16–68). Median waiting time and donor age have also increased from 40 to 513 days and from 27 to 44 years respectively (range 11–65). Donor cause of death is now primarily stroke, in contrast to head and brain injury in earlier years. Estimated median survival is 15.4 years (95% confidence interval 14.2–16.6) There is better survival throughout these years.

**Conclusion:**

Over the past 30 years, patient and donor demographics and underlying diseases have shifted substantially. Furthermore, the increase in waiting time due to lack of available donor hearts has led to a rise in the use of LVADs as bridge to transplant. Importantly, an improvement in survival rates is found over time which could be explained by better immunosuppressive therapy and improvements in follow-up care.

## Introduction

Orthotopic heart transplantation (HTx) has been an effective treatment for end-stage heart failure for many years and was performed in more than 120,000 patients worldwide up until 2015 [1]. Since 1967, when the first HTx was performed by Christiaan Barnard in South Africa, survival rates have increased significantly [2]. In the early days, this was mainly due to improvements in diagnosis and treatment of complications such as acute rejection [3]. These improvements were led by the introduction of the calcineurin inhibitors cyclosporine in 1980, and tacrolimus several years later, and the development of the bioptome, allowing diagnostic endomyocardial biopsies for the histological diagnosis of rejection. A systematic grading scale for the classification of rejection was also very important [4].

Nowadays, the main limitation of HTx is the lack of donor hearts worldwide. In the Netherlands the first HTx was performed in Rotterdam in 1984 and in Utrecht in 1985, after a long period of decision-making by the government. To date, around 100 patients are on the national waiting list, whereas approximately 45–50 patients are transplanted each year. This lack of donor hearts leads to prolonged waiting times. The limited availability of donor hearts is partly compensated for by left ventricular assist devices (LVADs), which are used to bridge patients with advanced heart failure until a donor heart becomes available. Interestingly, the improved durability of LVADs makes them suitable as a long-term alternative for HTx [3, 5, 6].

In this article we describe the demographics, indications, survival and donor characteristics over the past 30 years in patients who were transplanted at our centre.

## Methods

### Study design

This single-centre retrospective analysis included all patients ≥14 years of age who underwent orthotropic HTx at our centre from 1985 until 2015. Data were collected from a database containing prospectively registered heart transplantations performed after 1985, and missing data were collected from patient charts. For comparison over time, patients were grouped into six clusters by year of transplantation: (I) 1985–1989, (II) 1990–1994, (III) 1995–1999, (IV) 2000–2004, (V) 2005–2009 and (VI) 2010–2014.

### Screening, definition and in-house protocol

Patients were considered for HTx according to national guidelines, last updated in 2008 [7]. Briefly, indication for HTx is end-stage heart disease not amenable by more conservative measures. Since HTx is an intensive medical treatment, the patient must be willing, capable and emotionally stable to withstand the uncertainties likely to occur both before and after transplantation. Furthermore, the expected 1‑year mortality of the potential patient should exceed the 1‑year mortality after HTx, which is 10–15%. An estimation of the prognosis in patients with end-stage heart failure is difficult but can be estimated using, for instance, the Heart Failure Survival Score (HFSS) which consists of a combination of several non-invasive measures such as peak VO2, ejection fraction and intraventricular conduction delay, and the Seattle Heart Failure Model [8, 9].

Contraindications for HTx are defined as high pulmonary vascular resistance (PVR), active systemic infection, active malignancy, inability to comply with complex medical regimen, severe peripheral or cerebrovascular disease and irreversible dysfunction of another organ [6]. Nonetheless, these contraindications are generally not absolute but only temporary and have to be judged in relation to the clinical picture of the patient. As an example, irreversible elevated PVR increases the risk of right-sided failure of the transplanted heart. However, there is no absolute cut-off value so it has to be seen as an incremental risk factor.

After referral, the first step is optimisation of medical therapy after which patients undergo screening for contraindications. Eligibility of patients is assessed by a dedicated team consisting of at least a cardiologist trained in end-stage heart failure and transplantation, a cardiothoracic surgeon, and specialised nurses. According to the guidelines for HTx, patients are considered either: (1) not eligible for HTx, (2) a future candidate for HTx or (3) listed for HTx [7].

**Fig. 1:**
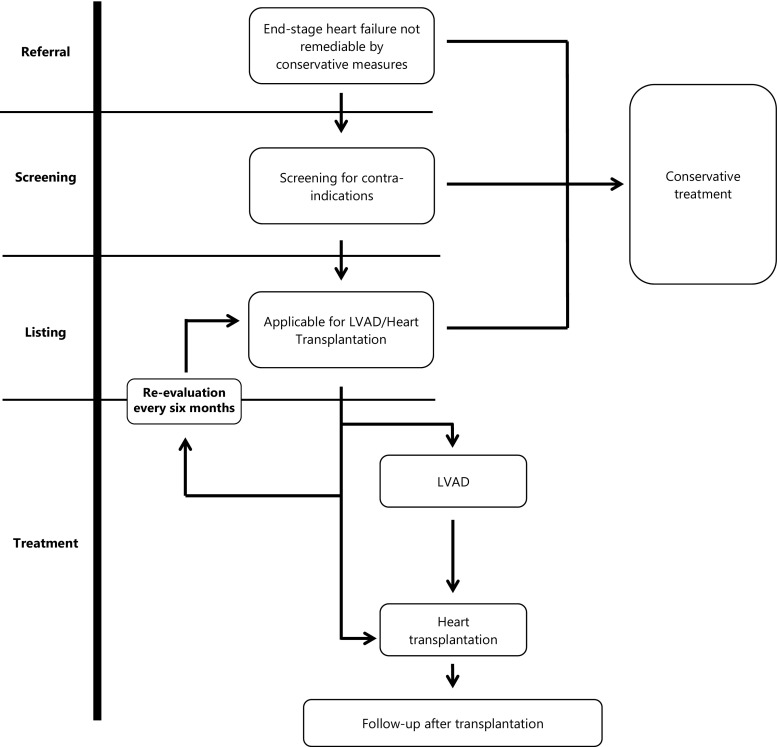
From referral to transplantation (*PHT* pulmonary hypertension, *HTx* heart transplantation, *CRTD* cardiac resynchronisation therapy device)

Patients on the waiting list, as well as the patients who were deemed too good for transplantation at prior evaluation, will be regularly re-evaluated given the dynamic nature of the clinical course (Fig. 1).

### Statistical analysis

Re-transplanted patients (*n* = 8) were listed as having one primary indication, and a secondary indication named ‘other’. Survival data were gathered using the hospital patient information system. Group comparisons were made using the chi-square test for categorical variables, the one-way ANOVA and post-hoc test for normally distributed continuous variables, and the Kruskal-Wallis or Mann-Whitney U test for non-normally distributed continuous variables when appropriate. Survival rates were calculated using the Kaplan-Meier method and tests for trends were performed using the log-rank test. Conditional survival curves were analysed for patients surviving the first year after HTx. Statistical significance was assumed at *p* < 0.05. Statistical analyses were performed using IBM SPSS version 21 for Windows (SPSS Inc., Chicago, Illinois, USA). Graphs and sub-analysis were performed using GraphPad Prism version 6.02 for Windows.

## Results

**Fig. 2:**
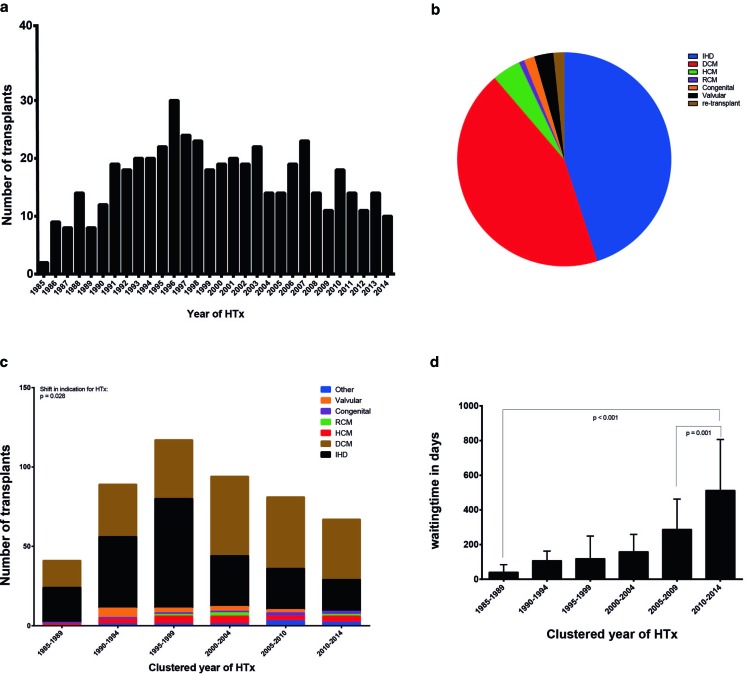
Number of transplants, indication for transplantation and median waiting time

This analysis includes 489 heart transplants in 481 patients in the UMCU from 1985 until 2015 (Fig. 2; Table 1).

**Table 1:** Characteristics of heart transplantation recipients

Patient characteristics *Averages presented as means ± SD or median (IQ) when appropriate*	HTx patients *n* = 489	Range or percentage
*Median age at transplantation (IQ)*	49 (IQ 39–56)	16–68
<20 years (*n*, %) 20–40 years (*n*, %) 40–60 years (*n*, %) >60 years (*n*, %)	10 126 306 47	2.0% 25.8% 62.6% 9.6%
Male *n*, %	372	76%
*Pretransplant diagnosis (n, %)*		
Non-ischaemic dilated CMP Ischaemic heart disease Hypertrophic CMP Restrictive CMP Congenital heart disease Valvular heart disease Re-transplant	220 214 21 4 8 14 8	45% 43.8% 4.3% 0.8% 1.6% (2.9%) 1.6%
Pretransplant BMI (±SD)	23.5 (±3.3)	13.7–34.9
Pretransplant PVR without intervention (±SD); (*n* = 471) Pretransplant PVR with intervention (±SD); (*n* = 18) Median pretransplant creatinine (IQ) (*n* = 483)	177 (±88) 230 (±92) 106 (IQ 89–127)	16-561 65–419 40–328
LVAD bridging, *n* (%) Median time with LVAD on waiting list in days (IQ); (*n* = 117)	120 266 (IQ 147–484)	25% (9–1384)
*Median waiting time for transplantation in days, (IQ), (n = 486)* 1985–1989 1990–1994 1995–1999 2000–2004 2005–2009 2010–2014	150 (IQ 48–301) 40 (IQ 16–84) 107 (IQ 41–162) 119 (IQ 43–249) 158 (IQ 56–259) 287 (IQ 119–463) 513 (IQ 257–806)	0–1688 0–262 3–653 0–559 1–1509 1–1235 1–1688

*SD* standard deviation, *IQ* interquartile range, *CMP* cardiomyopathy, *BMI* body mass index, *PVR* pulmonary vascular resistance, *LVAD* left ventricular assist device

### Recipient characteristics

Over time a gradual increase in numbers of transplantations per year can be seen, with a peak in 1996 and declining afterwards (Fig. 2a). Median age at HTx was 49 with an interquartile range (IQ) of 39–56 and has remained constant throughout the years. Over 60% of our patients were between 40–60 years of age at the time of transplant. Our cohort was predominantly male (76%) with no significant change over time. Primary indications for HTx were non-ischaemic dilated cardiomyopathy (DCM) (220 patients, 45%) and ischaemic heart disease (214 patients, 44%), followed by hypertrophic cardiomyopathy (21 patients, 4.3%), acquired valvular disease (14 patients, 2.9%), congenital heart disease (8 patients, 1.6%), restrictive cardiomyopathy (4 patients, 0.8%) and re-transplants (8 patients, 1.6%) (Fig. 2c; Table 1). Comparing indication for HTx, a significant shift can be seen (*p* = 0.028) in the number of recipients, from ischaemic heart disease to DCM over the course of the groups (Fig. 2b; Table 1). Whereas only 40% of recipients were of DCM origin in the first 5 years, this indication now comprises 57% of cases. Ischaemic heart disease, however, decreased from 52 to 30% (Fig. 2b; Table 2). Eight patients have had re-transplantations due to primary graft failure of the first donor heart. BMI increased from 22 to 24 over the years (*p* = 0.01). Mean PVR did not show changes.

**Table 2:** Significant change in demographics from 1985 to 2015

Change in demographics from 1985–1990 to 2010–2015 *Averages presented as means ± SD or median (IQ) and p‑value when appropriate*	1985–1989	2010–2014	*P*-value
*Pretransplant diagnosis* Non-ischaemic dilated CMP Ischaemic heart disease Hypertrophic CMP Restrictive CMP Congenital heart disease Valvular heart disease Other	40.5% 52.4% 2.4% 2.4% 2.4% –% –%	56.7% 29.9% 6.0% 1.5% 3.0% –% 3.0%	0.028
Pretransplant BMI (±SD)	22 (±3.5)	24 (±3.7)	0.01
Median waiting time for transplantation in days (IQ) (*n* = 486)	40 (IQ 16–84)	513 (IQ 257–806)	<0.001
*Donor cause of death (n = 484)*			
Brain tumour (%) Stroke (%) Gunshot wound, (%) Suicide (%) Head and brain injury (%) Unknown (%)	0% 30% 0% 2% 61% 7%	6% 65% 1% 10% 18% 3%	<0.001

*SD* standard deviation, *IQ* interquartile range, *CMP* cardiomyopathy, *BMI* body mass index

LVAD implantation in our centre began in 1993, first on a small scale. In total 120 patients (25%) received LVAD implantation prior to HTx and given the low numbers in the early years, a significant increase (*p* < 0.0001) in use can be observed later on with a median of 133 in 1990–1994 to a median of 594 days in 2010–2014. The average LVAD support time was a mean of 364 ± 313 days, ranging from 9–1384 days.

### Waiting time to transplantation

Overall median waiting time for transplantation was 150 (IQ 48–301) days with a range of 0–1688 days. A significant (*p* < 0.001) increase in waiting time can be seen from a median of 40 days in 1985–1990 to 513 days in 2010–2014. Since the introduction of continuous flow LVADs in 2006, with proven longer durability, the waiting time has increased even further (*p* = 0.001) (Fig. 2d; Table 1).

### Survival

**Fig. 3:**
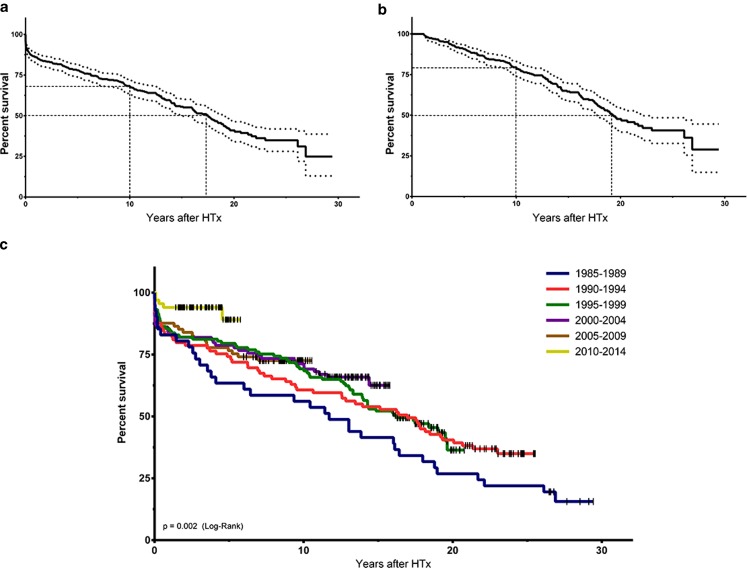
Overall survival, conditional survival and survival in groups

Kaplan-Meier data of total survival and conditional survival (those patients who survived the first year after HTx) are presented in Fig. 3. Median survival was 15.4 years (95% confidence interval 14.2–16.6) for the entire cohort, including 13 patients who have survived for over 25 years after HTx. There is a significant trend towards better survival when comparing the groups over time (Fig. 3).

### Donor characteristics

Median donor age was 40 [IQ 28–48] years for the whole cohort, but has increased significantly (*p* < 0.001) from 27 years to 44 years from 1985 to 2014. The oldest donor was 65 years; 207 (43%) of our donors were female and 280 (57%) were male (Table 3). The cause of death was mainly cerebral stroke (272, 57%) and head and brain injury (163, 34%). The remaining causes were brain tumours (14 = 3%), suicide (11, 2%), gunshot wounds (3, 1%) and 15 (3%) of unregistered cause. A significant (*p* < 0.001) shift in cause of death, however, can be observed. In the early years of HTx the major cause of death was head and brain injury (over 60% of donors), this has come down to 18% in recent years. The opposite holds true for stroke as a cause of death for donors (29 to 65%) (Table 2).

**Table 3:** Characteristics of heart transplantation donors

Donor characteristics *Averages presented as means ± SD or median (IQ) when appropriate*	*N*	Range or percentage
Donor age in years (IQ) (*n* = 482)	40 (IQ 28–48)	11–65
Male, *n *(%)	280	57%
*Donor cause of death (n = 477)*		
Brain tumour, *n* (%) Stroke, *n* (%) Gunshot wound, *n* (%) Suicide, *n* (%) Head and brain injury, *n* (%) Unknown, *n* (%)	14 272 3 11 162 15	3% 57% 1% 2% 34% 3%

*SD* standard deviation, *IQ* interquartile range

## Discussion

In this article we describe the demographics, indications and survival of HTx and donor characteristics over the past 30 years.

Firstly, addressing to demographic trends, we see that over the years DCM has replaced ischaemic heart disease as the main reason for HTx in our cohort. Worldwide this same trend can be observed [1, 7, 10–14]. One possible explanation might be the better treatment of coronary artery disease in itself, resulting in less patients with end-stage heart failure at an age that still allows HTx. Overall, the other characteristics of the recipients did not change very much over time; as can be expected, it concerns more men than women and the median age at which patients were transplanted was around 50. These figures are comparable with those from other European countries [12, 14].

Donor characteristics, however, did change dramatically from predominantly traumatic events as cause of death in the past to largely cerebrovascular events in more recent years, accompanied by a significant increase in donor age (median age 27 years in 1985, vs 44 years in 2014, with extremes to 65 years.) This change encompasses an entirely different risk profile of donors since hearts of older patients with stroke, by definition, have more vascular comorbidities, affecting not only the eligibility of the donor heart, but also result in an increased risk of coronary allograft vasculopathy after transplantation [15, 16]. With respect to donor age, Europe and especially the Netherlands completely diverge from the international data, as the median age of all cardiac donors used worldwide (including European data) is still only 35 years [1]. This has to be explained by the low mortality of traffic accidents in the Netherlands in comparison with other countries [3]. But without using those older donor hearts, almost no heart transplantations would be performed in the Netherlands.

Despite the significantly higher donor age, we demonstrate improved survival after HTx. This can be attributed to several factors. Apart from the availability of better immunosuppressive therapy and growing experience with this specific patient category in general, an important aspect is that all our follow-up is performed in-house and not elsewhere as in many other centres. Furthermore, international statistics are negatively biased by many smaller centres performing only a few transplantations per year and lacking this experience. This improved experience is also related to the treatment of complications such as cardiac allograft vasculopathy, renal failure and malignancies [3, 10]. Furthermore, because of the lack of donor hearts there is more stringent selection of recipients in comparison with other countries, potentially resulting in a younger transplantation cohort than reported by the International Society for Heart and Lung Transplantation (54 years) [1].

Another remarkable change over time is the use of LVADs as bridge to transplantation. This option was not available at the start of the program in 1985, but nowadays is an inseparable part of it. Due to the extremely long waiting time until transplantation, many patients deteriorate while on the waiting list. A large number of these patients can now be treated by mechanical circulatory support, using an LVAD as bridge to transplantation, which potentially causes deleterious displacement effects for the waiting time. The dilemma now conceived is an even longer waiting time as more patients survive until transplantation without an accompanying increase in donor hearts. But without the use of LVADs, many patients with acute heart failure would not have made it to transplantation or even to the waiting list at all. Clearly, implantation of an LVAD also implies perioperative risk but this outweighs the mortality of progressive acute heart failure by far [17]. Furthermore, it has to be realised that due to the improvements in technology and design the durability of LVADs has increased substantially, allowing the longer waiting time until transplantation, with a remarkable good quality of life and exercise tolerance [18, 19].

## Conclusion

Over the past 30 years, substantial differences can be noted in HTx. Patient demographics show a shift from ischaemic heart disease to DCM. The donor situation has completely changed from younger trauma victims to older patients dying from a cerebrovascular accident with a higher chance of pre-existing cardiovascular abnormalities. Due to the longer waiting time, an increasing number of patients have to be bridged to transplantation by a LVAD.

Despite these potentially adverse aspects, there is an improvement in survival rates which could be explained by better immunosuppressive therapy and improvements in follow-up care.
